# Alterations in gut microbiota and metabolomic profiles in acute stroke: insights into brain–gut axis dysregulation

**DOI:** 10.3389/fmicb.2025.1580231

**Published:** 2025-08-22

**Authors:** Jiajing Chen, Xiaodan Wu, Xintong Wang, Ruijia Yuan, Qi Zhu

**Affiliations:** ^1^Emergency Department, Central Hospital Affiliated to Shenyang Medical College, Shenyang, China; ^2^School of Clinical Medicine, China Medical University, Shenyang, China; ^3^School of Nursing, China Medical University, Shenyang, China

**Keywords:** acute stroke, gut microbiota, metabolomics, brain-gut axis, biomarkers

## Abstract

**Introduction:**

Acute stroke (AS) is a major public health issue globally, exhibiting high morbidity, disability rate, and mortality. Emerging research has demonstrated the critical roles of gut microbiota and its metabolites in pathogenesis, recovery, and prognosis of AS.

**Methods:**

In this study, we investigated alterations in gut microbiota composition and metabolomic profiles in AS patients using 16S rRNA sequencing and untargeted liquid chromatography-mass spectrometry (LC-MS) metabolomics technology.

**Results:**

The results revealed significant changes in gut microbiota diversity and community structure in AS patients compared with healthy controls. Notably, the abundance of anti-inflammatory microbiota was increased significantly, accompanied by elevated levels of certain metabolites, such as 6,9,12,15,18,21-tetracosahexaenoic acid and bufadienolide, while levels of urobilin and andrenid acid were significantly reduced. Network analysis further uncovered the significant diferences in microbiota-metabolite interactions between AS patients and healthy controls, indicating gut ecosystem disruption and functional dysfunction in AS.

**Discussion:**

This study sheds light on the mechanisms of brain-gut axis in AS, suggesting potential microbial and metabolite biomarkers, thus providing valuable insights into AS prediction and treatment.

## 1 Introduction

Acute stroke (AS) is a type of acute cerebrovascular disease that ranks the second leading cause of death and the third leading cause of disability worldwide, with an annual incidence of 24.9 million cases globally. It is characterized by high incidence, high disability rates, and mortality rates (Parr et al., 2017; Benjamin et al., 2018; Chidambaram et al., 2022). AS commonly occurs in elderly patients and can impair the central nervous system to varying degrees, resulting in dysfunction of autonomic nervous regulation (Kuo et al., 2020; Liu et al., 2024). Moreover, the resulting inflammatory response further aggravates brain tissue damage (Mozaffarian et al., 2015; Lee et al., 2023; Luo et al., 2024). Beyond the brain, stroke also induces systemic alterations, particularly affecting the gastrointestinal system. Many stroke patients experience varying degrees of injury to one or more peripheral organs, including the lung, heart, kidney, spleen, and gastrointestinal tract. Among these, gastrointestinal bleeding is a frequent and serious complication, particularly in patients with AS (Tuz et al., 2022; Wang et al., 2022).

The gut microbiota, as an essential component of host metabolism, is increasingly recognized as a vital “organ.” Emerging evidence has highlighted its involvement in the onset, progression and prognosis of AS, with gut dysbiosis contributing to elevated cerebrovascular risk and stroke pathogenesis (Peh et al., 2022; Battaglini et al., 2020; Yamashiro et al., 2021). Moreover, studies have identified a bidirectional communication system between the gut and the brain, known as the gut–brain axis. Within this framework, AS can lead to gut dysbiosis, while the composition of the gut microbiota may, in turn, influence the severity and progression of stroke (Zhai et al., 2023; Zhou et al., 2023; Shen and Mu, 2024; Shen et al., 2023). In stroke patients, gut microbiota dysbiosis is often characterized by an increased abundance of pro-inflammatory bacteria, such as *Prevotella* and Enterobacteriaceae, which can activate the Toll-like receptor 4 (TLR4) and nuclear factor kappa B (NF-κB) signaling pathways. This activation promotes the release of pro-inflammatory cytokines, including TNF-α, IL-6, and IL-1β, ultimately aggravating neuroinflammation and cerebral injury (Cryan et al., 2019; Arya and Hu, 2018; Wang X. et al., 2025). Concurrently, there is often a reduction in beneficial, anti-inflammatory bacteria such as *Faecalibacterium prausnitzii* and *Bifidobacterium*, which impairs the production of short-chain fatty acids (SCFAs) like butyrate and propionate. This reduction may decrease the activity of regulatory T cells (Tregs), leading to the overactivation of pro-inflammatory Th17 cells and exacerbating post-stroke inflammatory damage (Sun et al., 2020). Additionally, a decline in *Lactobacillus* may disrupt the synthesis of γ-aminobutyric acid (GABA), thereby compromising neuroprotective mechanisms (Henry et al., 2022; Durgan et al., 2019). Consistent with these findings, stroke-induced gut dysbiosis has also been observed in animal models, including mice and crab-eating macaques, where an overgrowth of Enterobacteriaceae and a decline in *Faecalibacterium* and *Lactobacillus* were associated with worsened neuroinflammation (Xu et al., 2021; Chen et al., 2019).

Metabolomics is a widely applied approach for profiling metabolites and offers unique advantages in identifying disease-specific mechanistic biomarkers (Bujak et al., 2016; Huang D. et al., 2023; Johnson et al., 2016). Changes in metabolites have been associated with dynamic alterations in gut microbiota (Wu et al., 2021; Fang et al., 2023). For example, gut microbiota can synthesize several nutritionally essential amino acids *de novo*, serving as a potential regulatory factor in maintaining amino acid homeostasis (Lin et al., 2017). Microbiota-derived metabolites have been shown to cross the intestinal mucosal barrier and enter the bloodstream, where they can traverse the blood–brain barrier and modulate microglial function (Del Rio et al., 2017; Erny et al., 2015). Compounds such as GABA, norepinephrine, dopamine, serotonin, tyramine, and tryptophan, produced by the gut microbiota, can directly influence brain cells or act on nerve fibers to facilitate gut–brain communication (Peh et al., 2022). Dysregulation of the gut–brain axis is increasingly recognized as a contributing factor to stroke risk and clinical outcomes, with specific microbiota-derived metabolites playing a critical role in stroke pathophysiology. However, the relationship between the gut microbiota and its metabolites in AS remains poorly understood, especially with respect to the identification of reliable biomarkers and elucidation of relevant metabolic pathways. In this study, a total of 20 patients with AS and 20 healthy controls were recruited. Fecal samples were collected and analyzed using 16S rRNA gene sequencing and untargeted liquid chromatography–mass spectrometry (LC-MS)-based metabolomics. This approach enabled us to characterize the gut microbiota and metabolite profiles in AS patients and explore their potential association with stroke onset. Our findings provide new insights into the gut–brain axis and suggest potential microbial and metabolomic biomarkers for the prevention, prediction, and treatment of stroke.

## 2 Materials and methods

### 2.1 Participant recruitment and samples

A total of 20 participants with AS were recruited from the Department of Neurology Central Affiliated Hospital of Shenyang Medical College between July to September 2022, aged 65.7 ± 8.4. Inclusion criteria were as follows: (a) Clinical presentation: patients exhibited one or more acute-onset of neurological symptoms, such as limb or facial weakness or numbness, sensory disturbances, dizziness, aphasia, dysarthria, dysphagia, ataxia, visual field defects or neglect, and cognitive impairment; (b) Neuroimaging: diagnosis was confirmed by a neurologist, based on the presence of acute lesions on diffusion-weighted imaging (DWI) via magnetic resonance imaging (MRI) or newly developed hypodense lesions on computed tomography (CT); (c) Time window: patients met the diagnostic criteria for AS and were admitted within 72 h of symptom onset (Neurology and Society, 2018; Liu et al., 2023).

Meanwhile, a total of 20 healthy participants (mean age: 61.5 ± 8.1 years) were recruited during the same period to serve as healthy controls. The inclusion criteria were: (a) Recent blood and biochemical tests within normal ranges; (b) No history of hypertension, diabetes, nephropathy, or cancer; (c) No recent use of medications, supplements, or dietary products known to alter gut microbiota or immune function; and (d) No clinical signs or history of inflammatory or infectious diseases.

The exclusion criteria for both groups were as follows: (a) Incomplete medical history or physical examination data; (b) Presence of conditions known to significantly alter the composition of gut microbiota or inflammatory status, such as hyperlipidemia or diabetes; (c) Recent use of medications, antibiotics, microecological agents, or health supplements known to affect gut microbiota composition or systemic inflammation; (d) Administration of ursodeoxycholic acid (UDCA) or bile acid chelators (e.g., cholestyreamine and cholestyrepo) within the previous month; and (e) Patients with cancer, mental illness, history of gastrointestinal surgery, hepatitis, liver cirrhosis, acute infection or acute cholecystitis/cholangitis within the last week.

To ensure the validity of the fecal sample collection and minimize potential confounding factors, all participants underwent thorough screening through clinical interviews and medical record reviews. Only those who met all inclusion criteria and were free from known interfering conditions were enrolled in the study. The fecal samples of AS patient group (T) were collected within 30 min of the patient's arrival at the emergency department. For the healthy group (C), fasting fecal samples were obtained at 8–10 a.m. following an overnight fast. All samples were immediately snap-frozen in liquid nitrogen and stored at −80 °C until further analysis.

This study was approved by the Ethics Committee of Central Affiliated Hospital of Shenyang Medical College [Sci-2024-008(01)]. Written informed consent was obtained from all participants prior to sample collection.

### 2.2 DNA extraction and 16S rRNA sequencing

Fecal samples from both groups were subjected to total microbiota genomic DNA extraction using FastPure Stool DNA Isolation Kit (MJYH, shanghai, China), following the manufacturer's instructions. The integrity of the extracted DNA was assessed by 1% agarose gel electrophoresis. DNA concentration and purity were determined using a NanoDrop2000 spectrophotometer (Thermo Scientific, USA). Qualified DNA was used as a template for PCR amplification of V3–V4 variable region of 16S rRNA gene, using upstream primer 338F (5′-ACTCCTACGGGAGGCAGCAG-3′) and downstream primer 806R (5′-GGACTACHVGGGTWTCTAAT-3′). The amplified PCR products were recovered and purified, and then quantified using Synergy HTX (Biotek, USA). A library was constructed using the NEXTFLEX Rapid DNA-Seq Kit (Bioo Scientific, Texas, USA) with the purified PCR products. Sequencing was performed on the Illumina NextSeq 2000 PE300 platform (Shanghai Majorbio Bio-Pharm Technology Co., Ltd., Shanghai, China).

### 2.3 Data analysis of 16S rRNA genomic sequencing

Paired-end raw sequencing data of the 16S rRNA gene were processed using qiime2 (v2024.5.0). Primer sequences 338F (ACTCCTACGGGAGGCAGCA) and 806R (GGACTACHVGGGTWTCTAAT) were removed from the forward and reverse ends using the qiime cutadapt trim-paired plugin. Subsequently, the primer-trimmed sequences were denoised, quality filtered, and processed to construct an amplicon sequence variant (ASV) feature table and representative sequences using the dada2 plugin in qiime2. The parameters used for denoising and quality control were –p-trim-left-f 0, –p-trim-left-r 0, –p-trunc-len-f 250, and –p-trunc-len-r 250.

The representative sequences obtained from the previous step were clustered *de novo* using the qiime vsearch cluster-features-*de-novo* plugin, with clustering performed at a 97% sequence similarity threshold to generate a 97% feature table and representative sequences. Taxonomic annotation of the filtered representative sequences was conducted using the qiime feature-classifier classify-sklearn classifier, aligning sequences to the SILVA v138 database. Chloroplast and mitochondrial sequences were removed from all samples during this process.

Alpha diversity indices, including evenness, faith_pd, observed features, and Shannon, were calculated using the qiime diversity core-metrics-phylogenetic plugin. Beta diversity was assessed based on Bray-Curtis and Jaccard distance metrics. Furthermore, functional features of the microbial communities were predicted using the FAPROTAX database (v1.2.10; Yang et al., 2022) and PICRUSt2 (v2.5.3; Ye and Doak, 2009).

### 2.4 Non-targeted metabolomics analysis based on LC-MS

Based on ASV clustering analysis of 16S rRNA gene amplicon sequencing (Supplementary Figure S1A) and inter-sample correlation assessment (Supplementary Figure S1B), six highly reproducible samples from both AS and healthy control groups were carefully selected for downstream metabolite extraction and profiling. A 50 mg fecal sample was ground using a 6 mm grinding bead, and 400 μL of extraction solution (methanol:water = 4:1, v:v) containing 0.02 mg/mL internal standard (L-2-chlorophenylalanine) was added to extract the metabolites. The samples were ground for 6 min using a refrigerated tissue grinder (−10 °C, 50 Hz) and then subjected to low-temperature ultrasonic extraction for 30 min (5 °C, 40 kHz). The extracted samples were left to stand at −20 °C for 30 min and then centrifuged at 13,000 g for 15 min at 4 °C. The supernatant was transferred to injection vials with inserts for LC-MS/MS analysis using the Thermo Fisher Scientific UHPLC-Q Exactive HF-X system (Shanghai Majorbio Bio-Pharm Technology Co., Ltd., Shanghai, China).

A quality control (QC) sample was prepared by pooling equal volumes of metabolites from all samples. During the analysis process, a QC sample was inserted every 5–15 samples to evaluate the reproducibility of the entire procedure.

After instrument analysis, the raw LC-MS data were imported into the metabolomics software Progenesis QI (Waters Corporation, Milford, USA) for baseline filtering, peak detection, integration, retention time correction, and peak alignment. This produced a data matrix containing retention time, mass-to-charge ratio (m/z), and peak intensity. Metabolite identification was performed by matching the MS and MS/MS spectral data against public metabolomics databases such as HMDB (http://www.hmdb.ca/), Metlin (https://metlin.scripps.edu/), and Majorbio's in-house database to obtain metabolite information.

### 2.5 Statistical analysis

For fecal community α-diversity, significance was assessed using the Kruskal-Wallis test. Principal coordinates analysis (PCoA) for β-diversity was conducted using the stats package in R (v4.4.1) to calculate explained variance, and visualized with the ggplot2 package. Hierarchical clustering was performed using the unweighted pair group method with arithmetic mean (UPGMA), and phylogenetic trees were generated using the ape package.

LEfSe analysis was conducted using the microeco package. LEfSe Cladograms, LDA score plots, and random forest analyses were visualized with ggplot2. Microbial interaction networks were constructed by calculating Spearman correlations using the Hmisc package and visualized using igraph and Gephi (v0.10.0).

Receiver operating characteristic (ROC) curves were generated using the pROC package to calculate the area under the curve (AUC) and the confidence intervals for AUC values. Anti-inflammatory microbial network diagrams were visualized with Cytoscape (v3.10.1). Differential analysis of microbial community functions was performed with a threshold of |log_2_FC| > 0 and *P* < 0.05 (Kruskal-Wallis test).

Principal component analysis (PCA) was performed using the prcomp function and visualized with ggplot2. Correlation heatmaps for metabolite samples and their correlation with microbiota were computed using Pearson correlation coefficients via the Hmisc package and visualized using the pheatmap and corrplot packages.

Orthogonal partial least squares discriminant analysis (OPLS-DA) was conducted using the ropls package. Differential metabolites were identified with thresholds of VIP > 1, |log_2_FC| > 1, and *P* < 0.05. These metabolites were further subjected to pathway enrichment analysis using the KEGG database via MetaboAnalyst (https://dev.metaboanalyst.ca/MetaboAnalyst/upload/EnrichUploadView.xhtml).

Canonical correspondence analysis (CCA) was performed using the vegan package to evaluate the relationships between gut microbiota and metabolites. Statistical significance was defined as *P* < 0.05.

## 3 Results

### 3.1 Gut microbiota composition was significantly altered in patients with AS

**Figure 1:**
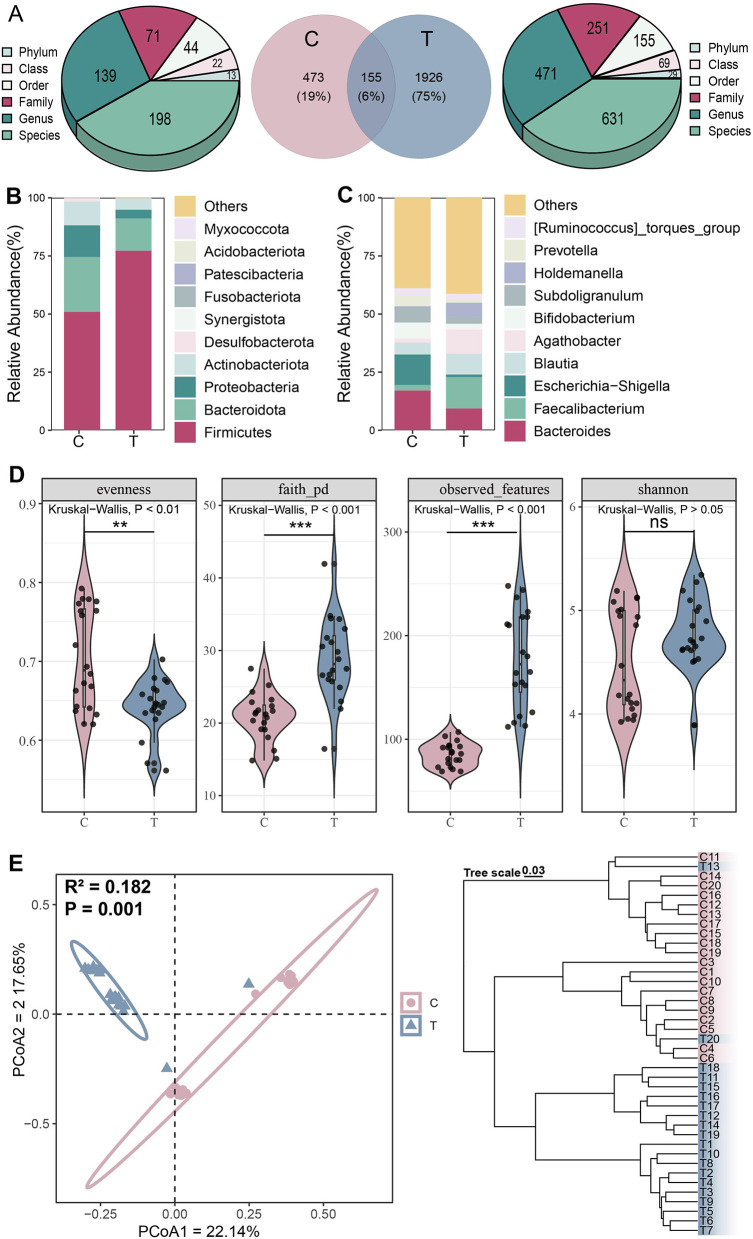
Microbial community structure analysis. **(A)** Distribution of amplicon sequence variant (ASVs) across different groups. **(B)** Phylum-level and **(C)** Genus-level species composition analysis. **(D)** α-diversity, including evenness, faith_pd, observed, and Shannon indices from left to right, Kruskal-Wallis test, *P < 0.05, **P < 0.01, ***P < 0.001, ns represents no significance. **(E)** β-diversity analysis, principal coordinate analysis (left) based on Jaccard distance (Adonis test: *R*^2^ = 0.182, *P* = 0.001) and hierarchical clustering (right) based on Bray-Curtis distance.

To investigate differences in gut microbiota structure between AS patients and healthy individuals, 16S rRNA sequencing was performed on the fecal samples of all participants. A total of 2,254 ASVs were identified, with 1,653 more ASVs detected in the T group than in the C group. ASVs in the T group were mainly derived from 29 phyla, 69 classes, 155 orders, 251 families, 471 genera, and 631 species, while in the C group, ASVs originated from 13 phyla, 22 classes, 44 orders, 71 families, 139 genera, and 168 species (Figure 1A). At the phylum level, Firmicuses, Bacteroidota, and Proteobacteria were dominant, with the relative abundance of Firmicutes higher in the T group than in the C group (Figure 1B). At the genus level, *Bacteroides, Faecalibacterium, Escherichia-Shigella*, and *Agathobacter* were predominant. Similarly, we observed that the relative abundances of *Faecalibacterium* and *Agathobacter* were higher in the T group than in the C group (Figure 1C).

Analysis of α-diversity revealed significantly higher phylogenetic diversity and coverage in the AS group compared with controls, while evenness was significantly lower (Kruskal-Wallis test, *P* < 0.05; Figure 1D). PCoA demonstrated clear separation between the C and T groups along the primary coordinate axes, indicating significant differences in gut microbiota composition between the two groups (Adonis test, *P* = 0.001). Hierarchical clustering based on Bray-Curtis distance further validated this distinction, with samples formed two distinct branches, demonstrating consistent microbial differences between the groups (Figure 1E). These findings collectively indicate that AS significantly altered the overall structure and diversity of the gut microbiota.

### 3.2 Marked gut microbiota dysbiosis observed in AS patients

**Figure 2:**
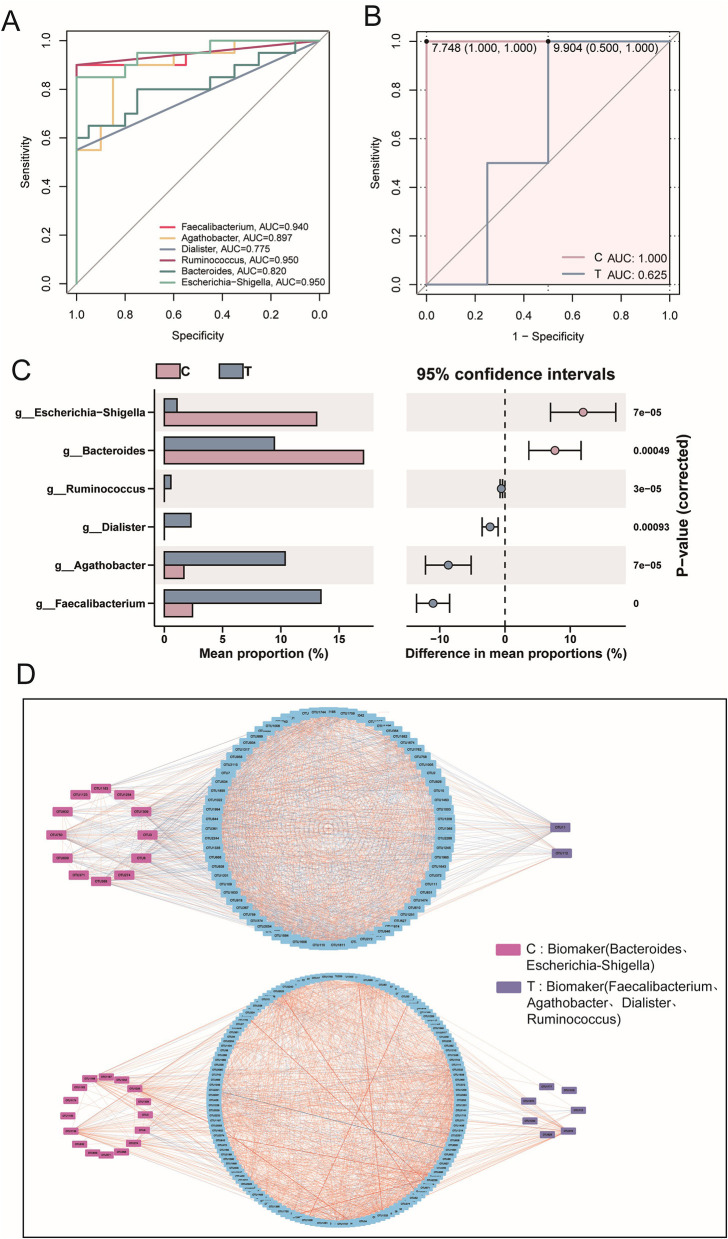
Signature species analysis. **(A)** Evolutionary tree diagram based on LEfSe analysis, where the circles radiating from the inner to the outer represent the taxonomic levels from phylum to species. Each small circle at different taxonomic levels represents a classification at that level, and the diameter of the circle is proportional to its relative abundance. **(B)** LDA scores for different species. **(C)** Random forest analysis at the genus level. **(D)** Species interaction analysis at the phylum level, based on Spearman correlation analysis. Species with |r| > 0.8 and *P* < 0.05 were selected for plotting. In the network diagram, blue lines represent negative correlations, red lines represent positive correlations, and different colored nodes represent different species. The size of the node represents the degree, indicating the number of interactions with other species.

Diversity analysis confirmed significant differences in microbiota between groups. To identify key microbial taxa, LEfSe analysis was performed to determine potential biomarkers. Healthy individuals exhibited a higher abundance of microbial taxa belonging to Proteobacteria and Bacteroidota, whereas AS patients showed a greater abundance of taxa concentrated in Firmicutes (LEfSe, *P* < 0.05, LDA > 2.0; Figure 2A). LDA scores highlighted the significance and importance of key microbial taxa in each group. The T group was found to be enriched in Firmicutes, including *Faecalibacterium* and *Blautia* (LEfSe, *P* < 0.05, LDA > 2.0). In contrast, healthy individuals were dominated by *Bacteroides* and *Escherichia-Shigella* (LEfSe, *P* < 0.05, LDA > 2.0; Figure 2B). Similarly, random forest analysis indicated that *Escherichia-Shigella* and *Megamonas* played a more significant role in classification within the healthy group. Meanwhile, *Barnesiella, Faecalibacterium*, and *Agathobacter* were key contributors in the AS patients, showing significantly higher abundances (Figure 2C).

At the phylum level, gut microbiota interaction networks revealed distinct topological differences. Healthy individuals exhibited a more stable and efficient network, characterized by fewer nodes (90) but a higher number of edges (1,760). Conversely, AS patients displayed a looser, less connected network, with more nodes (175) but fewer edges (1,525), indicating disrupted microbial interactions. Additionally, the average degree was lower in AS patients, indicating weaker interactions among microbial taxa compared with healthy individuals. Notably, AS patients exhibited additional interactions involving *Nitrospirota, MBNT15, Acidobacteriota, Gemmatimonadota*, and *Myxococcota* (Figure 2D). Collectively, these findings demonstrate that AS was associated with significant dysregulation of the gut microbiota, characterized by altered ecological structure and impaired microbial interactions.

### 3.3 Anti-inflammatory microbiota as characteristic biomarkers in AS patients

**Figure 3:**
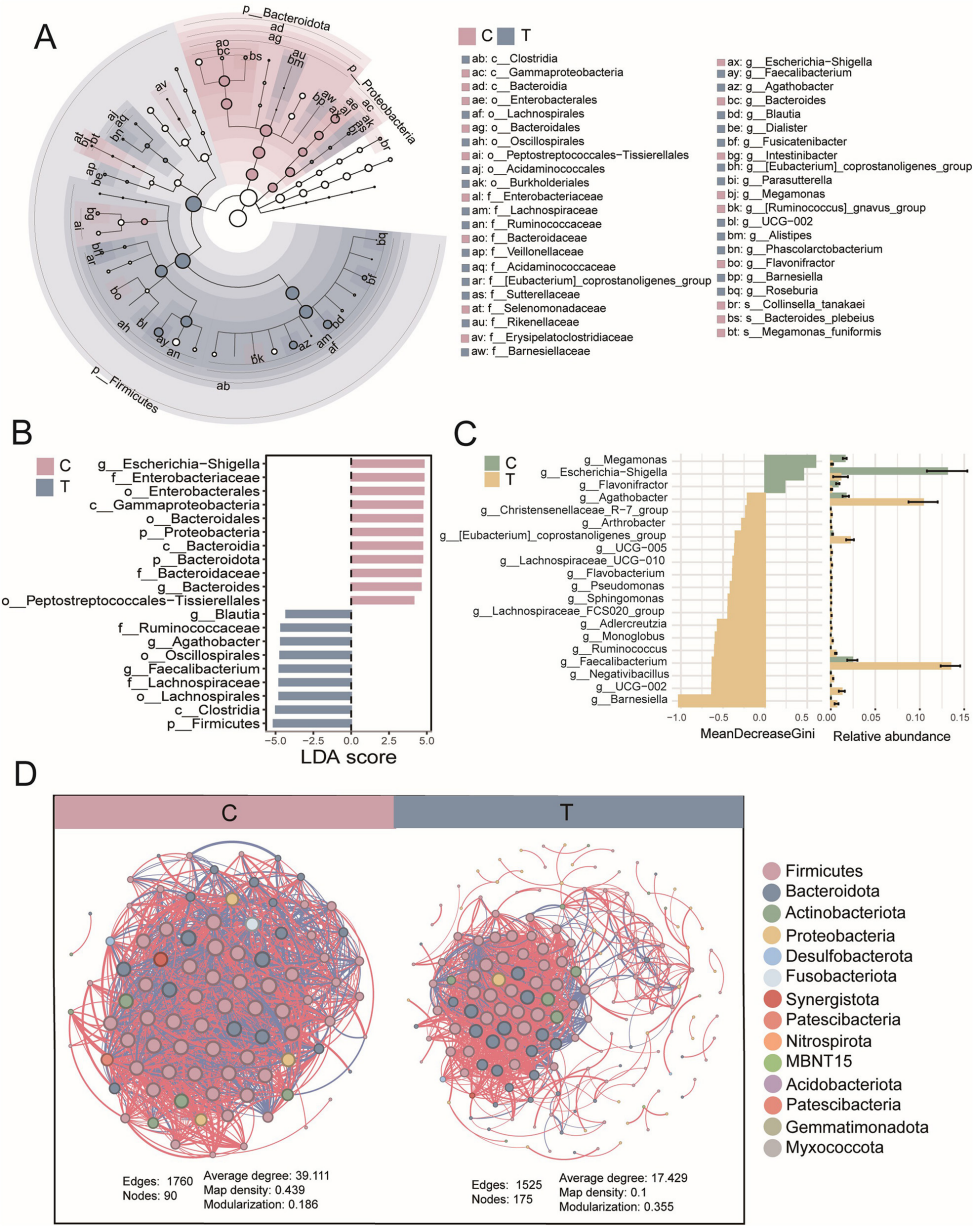
Biomarker statistics and interactions. **(A)** Receiver operating characteristic (ROC) curve analysis based on six signature genera to assess their ability to distinguish between the C group and T group. Each line of the same color represents a different species. **(B)** ROC curve analysis of signature genera between the C group (biomarkers, *n* = 4) and T group (biomarkers, *n* = 2). **(C)** Statistical differences in the relative abundance of biomarkers, evaluated using a *t*-test, with *P* < 0.05. **(D)** Associations between biomarkers and other genera (upper: C group; lower: T group), based on Spearman correlation analysis, with |r| > 0.8 and *P* < 0.05. In the network diagram, blue lines represent negative correlations, red lines represent positive correlations, red squares represent ASVs of biomarkers in the C group, purple squares represent ASVs of biomarkers in the T group, and blue boxes represent ASVs of other genera.

Based on prior LEfSe analysis, *Bacteroides* and *Escherichia-Shigella* were identified as key taxa in the healthy group, whereas *Faecalibacterium, Agathobacter, Dialister*, and *Ruminococcus*—recognized for their anti-inflammatory properties—were enriched in the disease group. To gain deeper insights into these microorganisms, further statistical and analytical evaluations were conducted to characterize their abundance patterns and potential functional implications in both groups. ROC curves and AUC values demonstrated strong discriminative power of all six potential microbial biomarkers in distinguishing the healthy and AS groups (Figure 3A). Additionally, the composite ROC curve constructed from the biomarkers showed high diagnostic sensitivity and specificity, reinforcing their potential as microbial indicators for distinguishing AS patients from healthy individuals (Figure 3B). Notably, biomarkers in the healthy group were significantly enriched in *Escherichia–Shigella* and *Bacteroides*, whereas the abundances of *Faecalibacterium, Agathobacter, Dialister*, and *Ruminococcus* were significantly elevated in AS patients (*T*-test, *P* < 0.05; Figure 3C). Subsequent interaction network analysis revealed distinct patterns between groups. In healthy individuals, microbiota interactions were predominantly negatively correlated, with 14 biomarker-associated nodes, only two of which belonged to T-group-specific taxa. Conversely, in AS patients, microbiota interactions shifted toward predominantly positive correlations, with 23 biomarker-associated nodes, including seven from T-group-specific taxa (Figure 3D). These findings suggest that the enrichment of anti-inflammatory microbiota may reflect a significant ecological shift in the gut microbiota of patients with AS.

### 3.4 AS may disrupt gut microbial ecology

**Figure 4:**
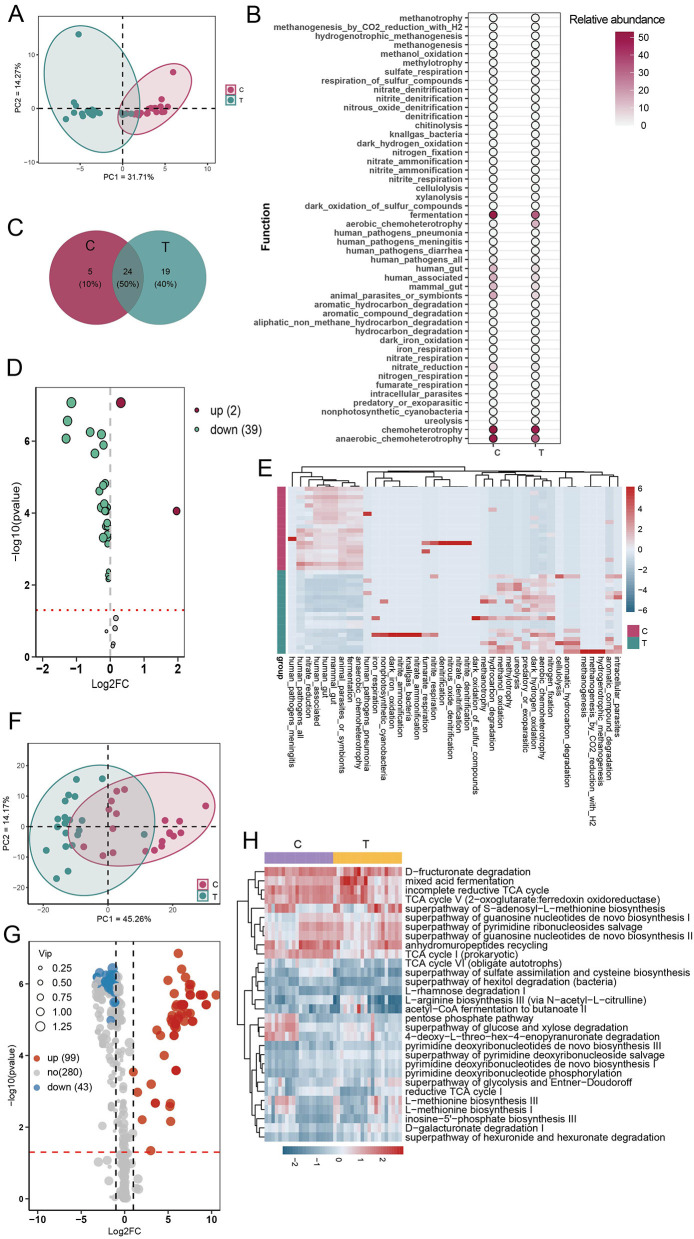
Functional prediction analysis of gut microbial communities using FAPROTAX and PICRUSt2. **(A)** Principal component analysis (PCA) of community functions based on FAPROTAX annotations. **(B)** Abundance statistics of all annotated community functions. **(C)** Analysis of shared and unique community functions between the C and T groups. **(D)** Volcano plot analysis of differential community functions (OPLS-DA analysis). **(E)** Heatmap analysis of differential community functions. **(F)** PCA of predicted metabolic pathways based on PICRUSt2 annotations. **(G)** Volcano plot based on OPLS-DA of predicted metabolic pathways using PICRUSt2. Pathways with VIP > 1 and FDR < 0.05 were considered significantly altered. Among them, pathways with an FC < 0.5 were defined as downregulated, while those with FC > 2 were considered upregulated. **(H)** Heatmap of the top 30 most abundant differentially expressed metabolic pathways.

To comprehensively assess microbial functional alterations in AS, we employed both FAPROTAX and PICRUSt2 for predictive functional profiling. PCA based on FAPROTAX-annotated functions showed a clear separation between AS patients and healthy controls (Figure 4A). A total of 48 functional groups were annotated, primarily involving chemoheterotrophy, fermentation, anaerobic chemoheterotrophy, animal parasites or symbionts, and human-associated functions. The gut microbiota of AS patients had 14 additional functional groups compared with healthy individuals (Figures 4B, C). Compared with the healthy group, the AS group showed a significant increase in the proportion of functions related to human pathogens (human_pathogens_all) and chemoheterotrophy. In contrast, the proportion of 39 functional groups associated with human gut, human-associated, and mammalian gut were significantly reduced in AS patients (Figures 4D, E). These findings suggest that AS may affect the gut ecology and host health by altering the functional composition of the gut microbiota. To further validate these findings, PICRUSt2 was used to predict KEGG-based metabolic pathways. Consistent with the FAPROTAX results, PCA based on these predicted metabolic pathways also revealed clear clustering between AS and control groups (Figure 4F). OPLS-DA identified 142 significantly altered pathways, among which 43 were significantly downregulated (VIP > 1, FDR < 0.05, FC < 2) and 99 upregulated (VIP > 1, FDR < 0.05, FC > 2) in the AS group (Figure 4G; see also Supplementary Table S1). Among the top 30 most abundant pathways, heatmap analysis showed a general trend of downregulation in the AS group, primarily involving carbohydrate metabolism (e.g., D-galacturonate degradation, TCA cycle variants), nucleotide biosynthesis, amino acid synthesis (e.g., L-methionine, L-arginine), and short-chain fatty acid production (Figure 4H). These results suggest that AS not only alters the taxonomic composition of gut microbiota but also profoundly disrupts its functional potential, particularly in energy metabolism and biosynthetic capabilities.

### 3.5 AS altered the composition and distribution of intestinal metabolites

**Figure 5:**
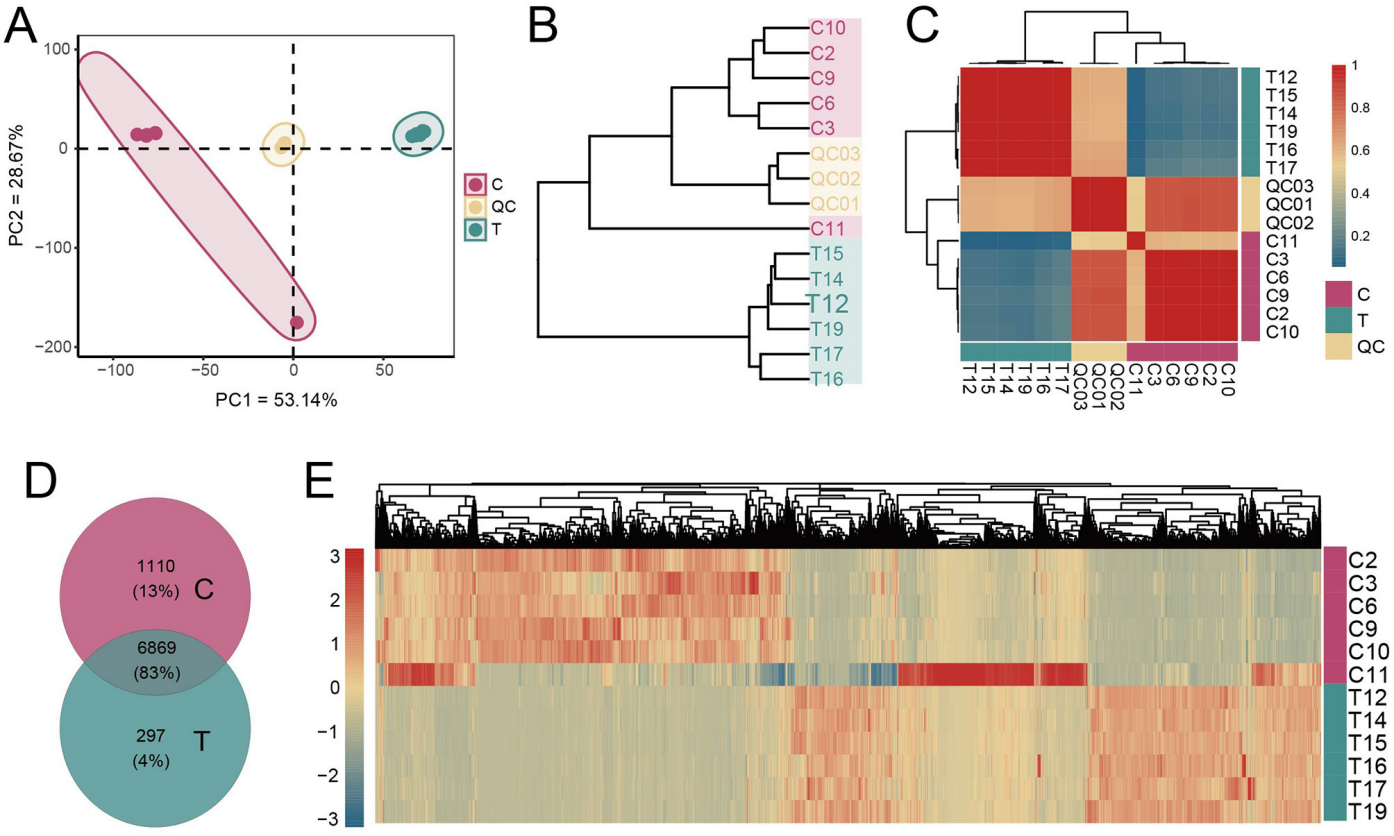
Metabolite composition analysis. **(A)** PCA of metabolite concentrations. **(B)** Clustering of samples based on the average clustering algorithm. **(C)** Correlation heatmap between samples (Pearson correlation). **(D)** Analysis of shared and unique metabolites between the C and T groups. **(E)** Heatmap analysis of the concentrations of all metabolites.

To investigate whether fecal metabolite composition was altered in AS patients, we conducted a comprehensive analysis of metabolite profiles. The evaluation of distribution patterns of all metabolites revealed that samples from the C group clustered closely together, showing high similarity (Pearson correlation, *r* > 0.8). Similarly, samples from the AS group (T group) also exhibited high similarity within their cluster (Pearson correlation, *r* > 0.8). This indicates a clear separation in metabolite distribution between healthy individuals and AS patients (Figures 5A–C). Across all samples, a total of 8,276 metabolites were detected. The C group contained 813 more metabolites than the T group, with 1,110 unique to the C group. In contrast, only 297 metabolites were unique to the T group. Additionally, heatmap visualization further highlighted the differential abundance of metabolites between groups (Figures 5D, E). These findings suggest that AS patients exhibited a distinct fecal metabolite profile compared with healthy individuals.

### 3.6 AS altered the composition of intestinal metabolites

**Figure 6:**
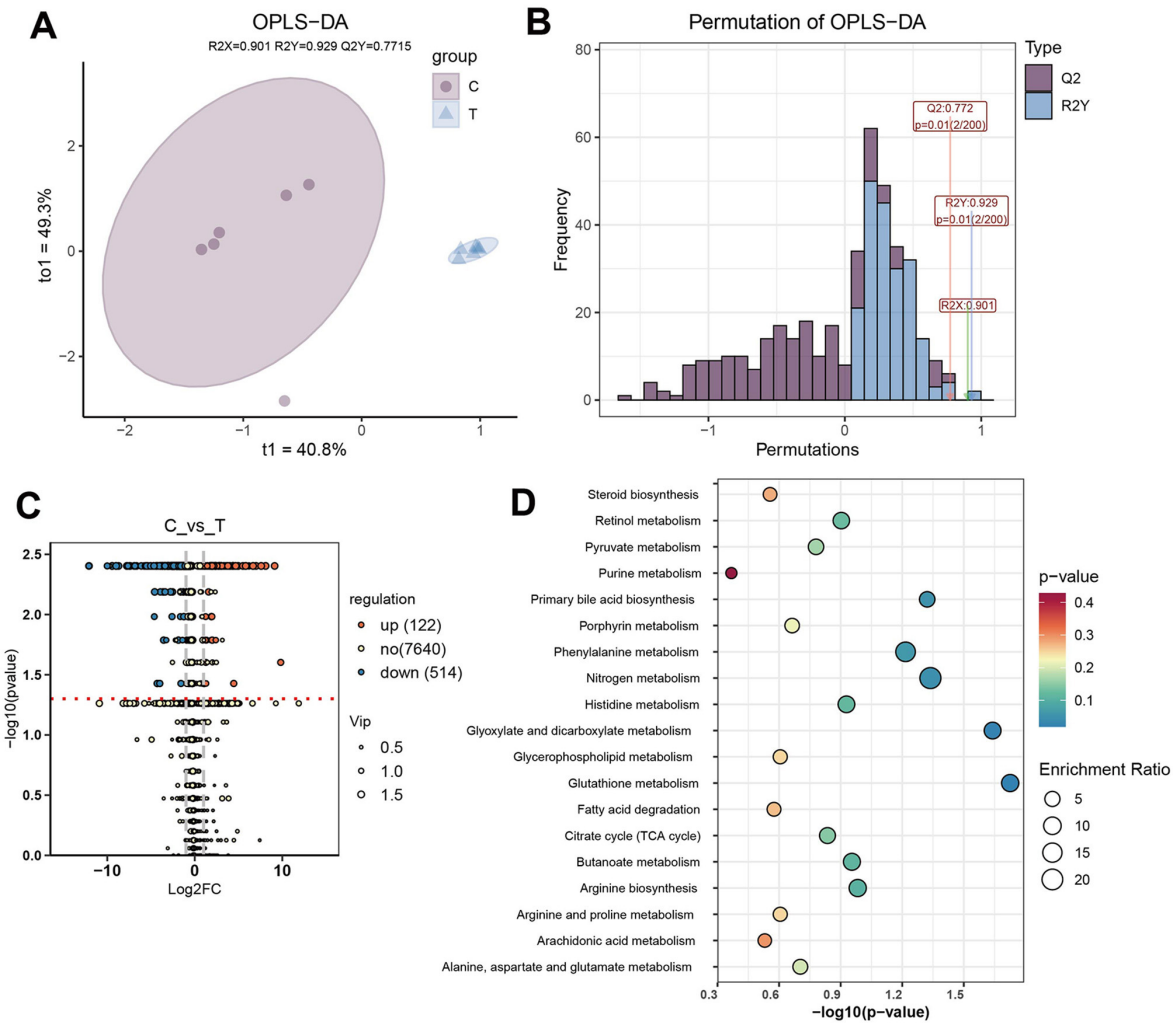
Differential metabolite analysis. **(A)** OPLS-DA plot based on the metabolic profiles of different groups. **(B)** Evaluation of the OPLS-DA model with 200 permutations. **(C)** Differential metabolite analysis between C and T groups (|log_2_FC| > 1, VIP > 1, and *P* < 0.05). **(D)** Enriched pathways of differential metabolites.

To investigate metabolic differences between healthy individuals and AS patients, OPLS-DA was performed on all detected metabolites. OPLS-DA revealed that, t1 and to1, accounted for 40.8% and 49.3% of the total variance, respectively. Clear separation between the C and T groups was observed, indicating that the model effectively captured and distinguished the group-specific metabolic profiles (Figure 6A). The reliability of the OPLS-DA model was validated using 200 permutation tests, yielding a *Q*^2^ value of 0.772 and an *R*^2^ value of 0.929, indicating strong predictive performance and explanatory capacity (Figure 6B). Differential metabolites were identified using the criteria |log_2_FC| > 1, VIP > 1, and *P* < 0.05. Compared with the C group, 122 metabolites were significantly upregulated in the T group, such as 6,9,12,15,18,21-tetracosahexaenoic acid, bufadienolide, and cucurbitacin D, while 514 metabolites were significantly downregulated, including urobilin, adrenic acid, and choldienic acid (Figure 6C, Supplementary Table S2). Enrichment analysis of the differential metabolites revealed that they were mainly enriched in pathways such as nitrogen metabolism, glutathione metabolism, glyoxylate and dicarboxylate metabolism, and phenylalanine metabolism (Figure 6D).

### 3.7 AS affected the interaction between gut microbiota and metabolites

**Figure 7:**
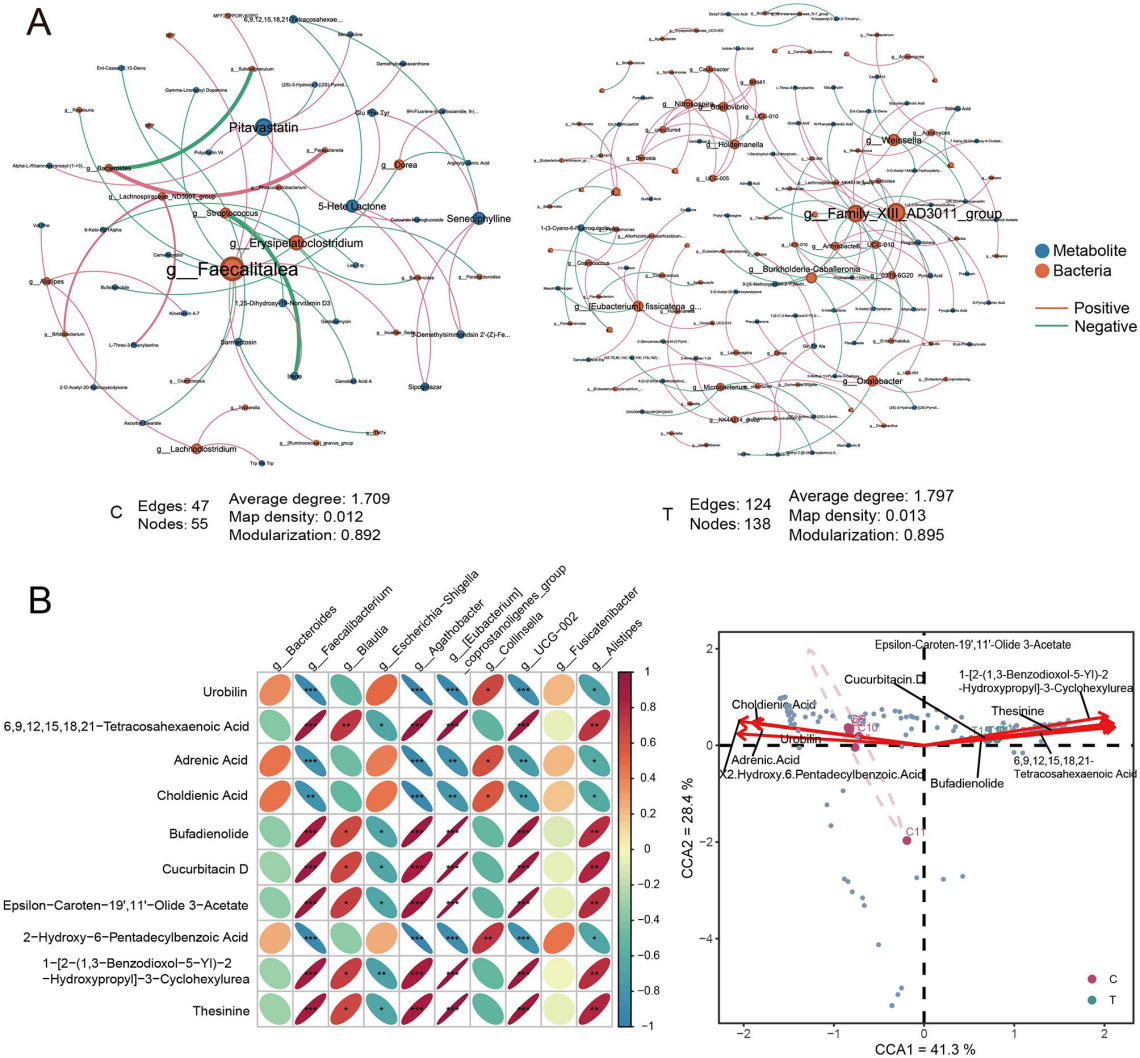
Correlation analysis of metabolites and prokaryotes. (A) Interaction network analysis of differential metabolites and all ASVs in C group (left) and T group (right), based on Spearman correlation analysis. Metabolites and species with *r* > 0.8 and *P* < 0.05 were selected for network construction. **(B)** Pearson correlation analysis between the top 10 metabolites by concentration and the top 10 species by relative abundance at the genus level. **(C)** Canonical correspondence analysis of genus-level differential species and the top 10 differential metabolites. Red arrows represent metabolites, blue points represent different species.

We further investigated the interactions between gut microbiota and metabolites in healthy individuals and AS patients. Interaction network analysis based on Spearman correlations (*P* < 0.05, *r* > 0.8) revealed distinct differences between the two groups. The C group exhibited 47 edges, 55 nodes, and an average degree of 1.709, whereas the T group exhibited a denser network with 124 edges, 138 nodes, and an average degree of 1.797, indicating more extensive microbe–metabolite interactions. Additionally, the central nodes in the interaction networks also differed between groups. Specifically, in healthy individuals, *Faecalitalea* and *Erysipelatoclostridium* emerged as key microbial hubs, with notable interactions involving metabolites such as Pitavastatin. In AS patients, microbial taxa such as *Family_XIII_AD3011_group* and *Weissella* displayed more associations with metabolites; however, the average node degree of metabolites was lower compared with that in the healthy group, indicating a more fragmented or specialized pattern of metabolite–microbe interactions (Figure 7A).

A correlation heatmap depicting the relationships between the top 10 differential metabolites and the top 10 abundant microbial genera showed significant patterns. Anti-inflammatory microbes, such as *Faecalibacterium* and *Agathobacter*, were positively correlated with metabolites such as 6,9,12,15,18,21-tetracosahexaenoic acid, bufadienolide, and cucurbitacin D (Pearson correlation, *r* > 0.9, *P* < 0.05). Conversely, these microbes were negatively correlated with metabolites like urobilin, adrenic acid, and 2-hydroxy-6-pentadecylbenzoic acid (Pearson correlation, *r* < −0.9, *P* < 0.05). These findings suggest that the metabolites may either be produced or modulated by these microbes, indicating the presence of potential symbiotic or mutualistic relationships between the gut microbiota and specific metabolites (Figure 7B). CCA of the top 10 differential metabolites and differential genera revealed a clear separation between the healthy and AS groups within the CCA space, reflecting significant shifts in the associations between microbial communities and metabolic profiles. Metabolites such as epsilon-caroten-9′,1′-olide 3-acetate and bufadienolide were closely associated with the AS group, suggesting that these metabolites were associated with stroke-specific microbial communities. Conversely, metabolites like choldienic acid and urobilin were linked to distinct microbial taxa in the healthy group (Figure 7C).

## 4 Discussion

Extensive research has demonstrated that AS leads to gut microbiota dysbiosis, and that the gut microbiota can modulate host physiology through the production of metabolites, thereby altering the composition and levels of gut metabolites (Zhao et al., 2023). Our study revealed significant changes in the gut microbiota and metabolites of patients with AS. The results indicated substantial alterations in the fecal microbiota community structure and metabolites in these patients. Specifically, the phylogenetic diversity and coverage of gut microbiota were significantly increased in AS patients. Anti-inflammatory microbes, such as *Faecalibacterium, Agathobacter, Dialister*, and *Ruminococcus*, which are key constituents of the gut ecosystem, were significantly more abundant than healthy participants. Previous research have identified *Faecalibacterium* as a core genus in the gut microbiota, notable for its production of SCFAs such as butyrate and propionate, which exhibit anti-inflammatory properties (Martín et al., 2023). Additionally, 122 metabolites, including 6,9,12,15,18,21-tetracosahexaenoic acid, bufadienolide, and cucurbitacin D, were significantly upregulated, whereas metabolites such as urobilin, adrenic acid, and choldienic acid were significantly downregulated. Notably, the abundances *Faecalibacterium* and *Agathobacter* were strongly linked to the upregulated metabolites.

AS induces significant alterations in the composition of gut microbiota (Singh et al., 2016). This dysbiosis may develop as a result of stroke-induced physiological and immunological changes, and in turn, altered gut microbiota can influence post-stroke inflammation and immune responses. Reduced diversity of Bacteroidota species and bacterial overgrowth have been identified as key features of post-stroke dysbiosis (Chen et al., 2019; Singh et al., 2016). In our study, the abundance of Bacteroidota was significantly reduced in stroke patients compared with healthy controls, while the abundance of Firmicutes was significantly increased. At the phylum level, such shifts in the overall gut microbiota have been implicated in providing neuroprotection during brain injury (Benakis et al., 2016). In a case-control clinical study on patients with large artery atherosclerotic ischemic stroke and transient ischemic attack, a reduced abundance of Bacteroidota was observed, accompanied by significant increases in microbial species richness, observed coverage, and phylogenetic diversity (α-diversity). The Shannon index showed a similar trend, but without significant differences (Yin et al., 2015). Our findings are consistent with these previous reports, reinforcing the notion that AS markedly alters gut microbiota composition and diversity.

In this study, compared with healthy individuals, the levels of gut microbiota such as *Faecalibacterium, Agathobacter, Dialister*, and *Ruminococcus* were significantly elevated in patients with AS, while the abundance of *Bacteroides* and *Escherichia-Shigella* was markedly reduced. Previous research has demonstrated that *Faecalibacterium* (Lopez-Siles et al., 2017), *Agathobacter* (Lv et al., 2024), *Dialister* (Downes et al., 2003), and *Ruminococcus* (Pal et al., 2021) produce butyrate and other SCFAs, which are involved in regulating glucose and fatty acid metabolism and exhibit anti-inflammatory properties (Parada Venegas et al., 2019). According to Huang et al., the gut microbiota profile of stroke patients showed significantly elevated levels of Firmicutes and Ruminococcaceae (Huang X. et al., 2023), which aligns with our findings. However, several other studies have reported a substantial reduction in anti-inflammatory genera such as *Anaerostipes, Ruminococcus*, and *Faecalibacterium*, alongside an enrichment of pro-inflammatory taxa such as *Enterococcus* and *Escherichia-Shigella* in stroke patients (Haak et al., 2021; Yamashiro et al., 2021; Li et al., 2019; Bonnechère et al., 2022), which contradict our observations. This discrepancy may be due to the acute-phase compensatory mechanism, where the body attempts to counteract the inflammatory response following a stroke. However, this hypothesis lacks direct supporting evidence. To address this, future studies should incorporate the analysis of inflammation markers, such as serum or fecal IL-6 and TNF-α, to validate the potential anti-inflammatory effects of the observed changes in gut microbiota. And longitudinal studies, either in animal models or clinical settings, are needed to track the dynamics of gut microbiota changes over time following a stroke. This will help to better understand the temporal relationship between gut microbiota changes and inflammatory responses, and to validate the proposed acute-phase compensatory mechanism.

These inconsistencies likely reflect the complexity and context-dependency of host–microbiome interactions, where the inflammatory potential of a specific microbial taxa may vary depending on host factors, microbial strain composition, and environmental influences. In the context of AS, this dynamic interaction may be particularly pronounced. AS represents the early phase of stroke, characterized by a sudden onset of symptoms within minutes to hours, requiring urgent medical intervention (Sacco et al., 2013). We hypothesize that such an abrupt event may induce rapid shifts in the gut microbiota. In response to the acute systemic inflammatory state, certain anti-inflammatory bacteria may increase in abundance as part of a compensatory mechanism to mitigate inflammation. Specifically, genera such as *Faecalibacterium* and *Agathobacter* might proliferate under stress to counteract systemic inflammation and maintain gut homeostasis. This hypothesis, however, warrants further investigation and experimental validation. It is also noteworthy that this study employed 16S rRNA amplicon sequencing targeting the V3–V4 regions, which typically provides taxonomic resolution only at the genus level, making it difficult to accurately distinguish species or strains (Tanes et al., 2024; Ames et al., 2017; Wang M. et al., 2025). As a result, critical strain-level differences may be overlooked, potentially limiting the depth of insight into the relationships between the gut microbiota and metabolic processes. Therefore, future studies should incorporate metagenomic sequencing and pure culture approaches to achieve higher-resolution identification and functional characterization of microbial strains.

Numerous studies have shown that anti-inflammatory biomarkers-derived SCFAs can penetrate the gut mucosal barrier, enter the bloodstream, cross the blood-brain barrier, and act directly on brain cells, inhibiting the production of pro-inflammatory cytokines (Erny et al., 2015; Vinolo et al., 2011). However, in our metabolomic analysis, SCFAs did not differ significantly between healthy controls and stroke patients. Instead, metabolites such as 6,9,12,15,18,21-tetracosahexaenoic acid, bufadienolide, cucurbitacin D, urobilin, adrenic acid, and choldienic acid exhibited significant alterations. These differential metabolites were primarily enriched in the nitrogen metabolism, glutathione metabolism, glyoxylate and dicarboxylate metabolism, and phenylalanine metabolism pathways, all of which are closely associated with oxidative stress, inflammatory responses, and energy metabolism. During stroke onset, the interruption of cerebral blood flow has been reported to result in insufficient oxygen and glucose supply, leading to impaired energy metabolism and exacerbated oxidative stress (Qin et al., 2022). Alterations in nitrogen and glutathione metabolism likely reflect a defensive response to oxidative damage. Meanwhile, dysregulation of glutathione metabolism may paradoxically contribute to neuronal injury (Shin et al., 2020; Pluta and Januszewski, 2023). In addition, abnormalities in phenylalanine metabolism have been associated with neuroinflammation and neurotoxicity, potentially exacerbating post-stroke neurological dysfunction (Wang et al., 2020, 2024). These shifts in gut-derived metabolites suggest that the gut–brain axis plays a critical role in the pathophysiology of AS, possibly through the modulation of systemic inflammation and neuroprotective mechanisms (Yamashiro et al., 2017).

The observed elevation of long-chain polyunsaturated fatty acids (PUFAs) in this study, such as 6,9,12,15,18,21-tetracosahexaenoic acid, along with steroidal and triterpenoid metabolites, may reflect a compensatory regulatory response to inflammation and oxidative stress. This finding aligns with recent reports highlighting the neuroprotective roles of lipid metabolism in stroke (Shin et al., 2020; Wang et al., 2020; Peesh et al., 2025). Notably, *Faecalibacterium* and *agathobacter* were found correlated positively with 6,9,12,15,18,21-tetracosahexaenoic acid, bufadienolide, and cucurbitacin D. 6,9,12,15,18,21-tetracosahexaenoic acid, a long-chain PUFA, is closely linked to lipid metabolism and inflammatory responses. It has been shown to inhibit NF-κB and COX-2 signaling pathways, thereby reducing the production of pro-inflammatory cytokines such as TNF-α and IL-6 (Metherel et al., 2017; Lacombe et al., 2018). Additionally, it promotes the generation of specialized pro-resolving mediators (SPMs) like resolvins, which facilitate the resolution of inflammation (Calder, 2013). Bufadienolide, a steroidal endotoxin-like compound, participates in steroid metabolism and inflammatory signaling pathways, such as TLR signaling. It has been reported to inhibit the TLR4/NF-κB pathway, reducing the release of pro-inflammatory cytokines like IL-1β and TNF-α (Deng et al., 2020). Furthermore, bufadienolide can induce apoptosis in overactivated immune cells, thereby mitigating excessive inflammatory responses (Chen et al., 2023). Cucurbitacin D, a triterpenoid compound, is involved in apoptosis and anti-inflammatory pathways such as the MAPK/ERK pathway. It has been shown to inhibit the MAPK/ERK signaling cascade, leading to reduced expression of pro-inflammatory cytokines like IL-6 and COX-2 (Song et al., 2013). Moreover, cucurbitacin D can induce cell cycle arrest in rapidly proliferating immune cells, further contributing to its anti-inflammatory effects (Mehdi Üremiş et al., 2022).

Based on the above findings, we hypothesize that anti-inflammatory gut microbiota in AS patients may participate in the regulation of inflammatory responses by modulating the production of specific metabolites such as 6,9,12,15,18,21-tetracosahexaenoic acid, bufadienolide, and cucurbitacin D. These microbial–metabolite interactions may also represent potential therapeutic targets for future interventions. However, current research on the anti-inflammatory mechanisms of commensal bacteria, particularly those involving SCFA secretion, remains limited. Moreover, our study only provides preliminary correlation analyses between gut microbiota and metabolites. To further validate these initial observations, future studies should integrate animal models to explore the causal roles of *Faecalibacterium* and *Agathobacter* in regulating the production of 6,9,12,15,18,21-tetracosahexaenoic acid, bufadienolide, and cucurbitacin D, and their subsequent impact on inflammatory responses. For example, fecal microbiota transplantation or targeted bacterial interventions in AS animal models, combined with metabolomics and inflammatory marker analyses, could help elucidate the causal mechanisms underlying microbe–metabolite–inflammation interactions (Agus et al., 2021; Xu et al., 2022; Su et al., 2025). Additionally, *in vitro* cell culture experiments may be utilized to elucidate the specific inflammatory signaling pathways modulated by these metabolites.

In addition to the aforementioned points, this study has several general limitations. First, the sample size was relatively small. Moreover, samples for metabolomics analysis were further selected using stratified random sampling, which may have introduced selection bias. Although efforts were made to ensure representativeness, the limited sample size may still affect the statistical power and generalizability of the findings. Second, while the study integrated microbiota and metabolite data, it was cross-sectional in design, preventing the establishment of causal relationships. Third, medications such as antiplatelet and statin drugs, commonly prescribed to stroke patients, have been reported to influence gut microbiota (Yamashiro et al., 2017). Similarly, post-stroke dietary changes, which often involve increased fiber intake and reduced fat consumption, can also affect gut microbiota composition (Sonnenburg and Bäckhed, 2016). However, a notable limitation of this study is the lack of detailed pre-admission medication and dietary information. To validate and expand upon the current observations, future studies should employ larger, well-controlled cohorts and longitudinal designs.

## 5 Conclusion

In this study, we employed 16S rRNA sequencing and untargeted metabolomics to investigate alterations in the gut microbiota and metabolite profiles of AS patients. Compared with healthy individuals, AS patients exhibited a significantly increased abundance of anti-inflammatory microbes. Notably, no significant differences in SCFA levels were detected between the two groups. Instead, metabolites such as 6,9,12,15,18,21-tetracosahexaenoic acid, bufadienolide, and cucurbitacin D were markedly elevated in AS patients and showed strong positive correlations with the enriched anti-inflammatory microbiota. Our findings indicate that gut dysbiosis in AS patients is closely associated with changes in specific metabolites. This intricate microbe-metabolite-host interaction likely reflects a unique gut metabolic adaptation mechanism in stroke patients. These results provide new insights into the role of the gut-brain axis in AS and propose potential microbial and metabolic biomarkers for stroke diagnosis and treatment. Future studies should further validate the biological functions of these metabolites and their specific relationships with stroke prognosis, thereby laying the groundwork for developing microbiota-based precision therapeutic strategies.

## Data Availability

The metabolomics data generated and analyzed in this study have been deposited in the MetaboLights repository under accession number MTBLS12822 (https://www.ebi.ac.uk/metabolights/MTBLS12822). The 16S rRNA sequencing data are publicly available in the NCBI Sequence Read Archive (SRA) under accession number PRJNA1302100 (https://www.ncbi.nlm.nih.gov/bioproject/PRJNA1302100).
