# Supplementary material for: Alterations in gut microbiota and metabolomic profiles in acute stroke: insights into brain–gut axis dysregulation
Source: Front Microbiol. 2025 Aug 22;16:1580231. doi: 10.3389/fmicb.2025.1580231 (PMC12411451; doi:10.3389/fmicb.2025.1580231)
Supplement: Supplementary file 3 [file Data_Sheet_2.docx]

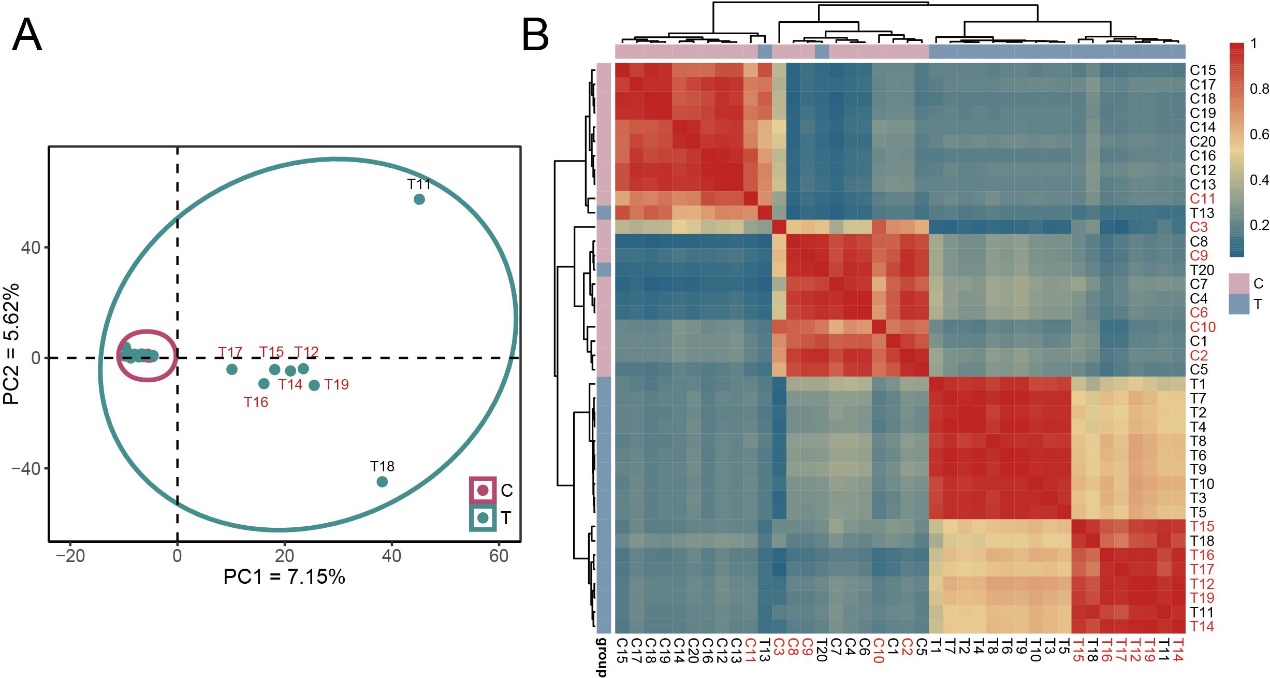


**Fig. S1** Reproducibility assessment of samples based on 16S rRNA gene amplicon ASV data. **A.** Principal component analysis (PCA) of 16S rRNA gene amplicon ASV data, with ellipses indicating 95% confidence intervals. **B.** Pearson correlation analysis among samples.

**Table S1** Differential analysis of metabolic pathways based on PICRUSt2 annotations using OPLS-DA.

**Table S2** Differential metabolites statistics.
